# Supplementary material for: Inferring fine-grained migration patterns across the United States
Source: Nat Commun. 2025 Dec 26;17:1265. doi: 10.1038/s41467-025-68019-2 (PMC12868740; doi:10.1038/s41467-025-68019-2)
Supplement: Supplementary file 1 — Supplementary Information [file 41467_2025_68019_MOESM1_ESM.pdf]

Inferring fine-grained migration patterns across the United States

Gabriel Agostini[1], Rachel Young[2], Maria Fitzpatrick[3], Nikhil Garg[1], Emma Pierson[2*]

[1]Cornell Tech, New York City, NY, United States
[2]University of California, Berkeley, Berkeley, CA, United States
[3]Cornell University, Ithaca, NY, United States

[*]**Corresponding author:** emmapierson@berkeley.edu

List of Supplementary Items

Additional details on validations . . . **2**
- Validating the estimation procedure using held-out data . . . 2
  - Supplementary Table 1 . . . 2
- Validating the harmonization procedure with semi-synthetic data . . . 3
  - Supplementary Figure 1 . . . 4
- Related work documenting biases in the Infutor data . . . 5
- Assessment of additional demographic biases . . . 5
  - Supplementary Figure 2 . . . 5
  - Supplementary Figure 3 . . . 6

Additional analyses of national migration . . . **8**
- Out-migration rates by CBG type . . . 8
  - Supplementary Table 2 . . . 8
- Additional statistics on migration homophily . . . 8
  - Supplementary Figure 4 . . . 8
- Additional statistics on economic mobility in migration . . . 9
  - Supplementary Figure 5 . . . 9
- Additional statistics on moving distance . . . 10
  - Supplementary Figure 6 . . . 10

Additional case studies of local migration patterns . . . **11**
- Case Study: destinations of New York City outmovers . . . 11
  - Supplementary Figure 7 . . . 11
- Case Study: New York City Housing Authority . . . 12
  - Supplementary Figure 8 . . . 12

Privacy Protections . . . **14**

Additional details on validations

Here we provide additional details on MIGRATE validations. We include additional details on our held-out data validations; a semi-synthetic data validation where we verify our method reconstructs a known ground-truth matrix; details on related work documenting biases of Infutor, which is the subject of our Figure 3; and additional socioeconomic and demographic dimensions on which we assessed biases, including undocumented immigrants.

Validating the estimation procedure using held-out data

**Pearson Correlations**		**MIGRATE**	**CBG Held-Out**	**State Held-Out**
**Population counts**	State	1.000	1.000	1.000
	County	1.000	1.000	1.000
	Tract	0.997	0.888	0.998
	CBG	0.996	0.856	0.997
**Population flows**	State	1.000	1.000	1.000
	County	1.000	1.000	1.000
**Population flows (area movers only)**	State	0.998	0.998	0.895
	County	0.957	0.956	0.949
**Population flows (area stayers only)**	State	1.000	1.000	1.000
	County	1.000	1.000	1.000
**In-migration**	State	0.998	0.998	0.885
	County	0.984	0.983	0.963
**In-migration rate**	State	0.987	0.987	0.663
	County	0.715	0.705	0.629
**RMSE Reductions (%) from raw Infutor**		**MIGRATE**	**CBG Held-Out**	**State Held-Out**
**Population counts**	State	100.00	100.00	100.00
	County	100.00	100.00	100.00
	Tract	85.88	13.46	88.13
	CBG	83.60	9.08	85.09
**Population flows**	State	89.46	89.40	80.87
	County	90.65	90.70	75.24
**Population flows (area movers only)**	State	87.46	87.06	4.08
	County	42.34	41.30	4.65
**Population flows (area stayers only)**	State	89.46	89.40	81.03
	County	91.01	91.08	75.59
**In-migration**	State	90.93	91.11	7.22
	County	68.92	68.19	6.55
**In-migration rate**	State	87.30	87.33	4.89
	County	51.75	50.91	0.73

Supplementary Table 1: Validation of estimates in held-out data settings compared to Census data. The full model validations are repeated for comparison. For in-migration rates, metrics are weighted by populations to account for disparate county and state sizes. As noted in the main text, perfect correlations between MIGRATE and all flows or stayers are partially driven by variation in area size and the predominance of non-mover counts among flows. RMSE reduction values are average reductions across all years with overlapping ground-truth data releases, and raw Infutor is rescaled to account for trivial error reductions.

We verify that our estimation procedure yields highly correlated estimates with each Census data source even when it is removed from the datasets used for estimation. For example, we remove CBG-level Census populations from our estimation datasets, and verify that our resulting estimates remain highly correlated with CBG-level data. Specifically, we conduct our full battery of validations on two additional sets of migration estimates: "CBG held-out", which is estimated without the CBG-level population data (i.e. $E^{(t)}$ was not scaled to match ACS 5-year CBG populations); and "State held-out", which is estimated without the state-level flow data (i.e. $E^{(t)}$ was not scaled to match ACS 1-year state-to-state flows nor state mobility rates). Supplementary Table 1 reports values for the Pearson correlation across all estimates and ground-truth as well as relative error reduction from Infutor. Values for the original MIGRATE estimates, reported in Figure 2, are reproduced for easy comparison.

The CBG held-out and State held-out estimates remain highly correlated with all Census datasets (ρ above 0.629); they also reduce RMSE relative to Infutor. Unsurprisingly, both the correlations and error reductions are smaller than those achieved by our full MIGRATE estimates, which make

use of all sources of Census data. Together, these results illustrate both that (a) our estimation pipeline generalizes well to held-out data and that (b) all datasets used in the training help improve concordance with Census data, justifying their inclusion.

Validating the harmonization procedure with semi-synthetic data

We further validate our harmonization procedure using semi-synthetic data, a standard check for statistical methods when ground-truth data is lacking [1–3]. Specifically, we assess how well our harmonization procedure can recover a known CBG-to-CBG flow matrix, given only a perturbed version of that matrix and the same set of marginal constraints available in our main analysis (e.g., CBG-level populations).

To generate realistic semi-synthetic data, we use the MIGRATE matrices as the ground-truth CBG-to-CBG flow matrices M, since these are likely more realistic than purely synthetic data generated from stylized models. These ground-truth matrices M are not used as input to our harmonization method, but only as validation. From M, we produce the perturbed CBG-to-CBG flow matrix E by adding multiplicative noise; we describe this process further below. We also compute the same marginals of M that are available in our main analysis: specifically, CBG populations; state-level counts of movers and non-movers; state-to-state flows; and county populations. We use these marginals and the perturbed matrix E as inputs to our harmonization method. We assess how well our method recovers the ground-truth matrix M using the same metrics described in the main text (reduction in RMSE relative to using E alone, and Pearson correlation).

To thoroughly test the robustness of our method, we consider two processes for generating E from M. We now describe these processes.

Harmonizing in the presence of structured multiplicative noise

Our harmonization procedure assumes that the difference between the ground-truth matrix M and the perturbed matrix E can be described by a series of multiplicative scalings with a given structure (e.g., CBG-level scalings on each row) as described in the Methods Section. We thus first test how well our harmonization procedure recovers the ground-truth matrix if the perturbation does indeed match that structure. Specifically, we draw independent Log-Normal random variables that multiply (1) each row of M (mirrors CBG population fitting), (2) each group of diagonal entries within the same state (mirrors state non-movers fitting), (3) each group of off-diagonal entries in columns within the same state (mirrors state movers fitting), (4) each group of entries within the same state-to-state pair (mirrors state flows fitting) and (5) each group of rows within the same county, and each group of columns within the same county (mirrors county population fitting). We draw the Log-Normal noise using standard deviations τ ranging from 0.05 to 0.20. We choose the upper bound on this range because it exceeds the level of variation we observe in real-world data. Specifically, across states (Figure 3**a**), the biases between ground-truth and Infutor populations have standard deviation 0.11; across counties, the standard deviation is 0.19, both exceeded by our upper bound of $\tau = 0.20$. (We do verify, however, that results remain robust up to much larger τ values of 1.00).

We find that our harmonization method performs very strongly in this setting. For all standard deviations of noise, our inferred matrix achieves Pearson correlations above 0.99 either on the full CBG-CBG matrix or when restricting to CBG-CBG movers. The harmonized matrix also reduces RMSE (relative to the original perturbed matrix E) by an average of 97.7% across all years and noise levels (sd 0.06%), and by 91.9% when restricting to off-diagonal entries (sd 0.67%). These RMSE reductions remain consistently strong across the range of noise scales. The small residual error is an expected result of the optimization procedure; for example, we only fit once to CBG-level marginals to avoid overfitting, so are not guaranteed to match these marginals.

Harmonizing in the presence of real-world bias and independent noise

We next assess the performance of our harmonization procedure when the perturbation does not match the structure it assumes, but consists both of real-world bias and independent noise. Specifically, we produce the perturbed matrix E from M as follows:

$$E_{ij} = M_{ij} \cdot \exp\left\{b \cdot (w_i + w_j) + \sigma \cdot Z_{ij}\right\}$$

where w_i is the z-scored share of the white population in CBG i (capturing real-world biases, as shown in Figure 3**c**) and Z_{ij} are i.i.d. standard normal variables. The parameters b and σ control

the relative scales of the bias and the independent noise, respectively. We assess performance across a range of b and σ, choosing b to represent a realistic level of real-world bias based on our results in Figure 3**c**, and noise scales σ up to 0.20, as above.

We would expect high levels of independent noise to make it very challenging to recover the off-diagonal entries of the ground-truth matrix. This is because the diagonal entries of the migration matrix are much bigger than the off-diagonal entries (since most people do not move); thus, the scaling factors learned by the harmonization procedure will be dominated by the diagonal entries. This is generally reasonable: if we want to estimate bias in the movers from a CBG, the bias of the entire CBG population likely serves as a good proxy. However, this logic fails under independent noise, since noise in the diagonals provides no information about noise in the off-diagonals. Importantly, the challenge of correcting for independent noise applies not just to our harmonization procedure, but to any correction procedure, since correcting such noise would require information about the ground truth for each entry.

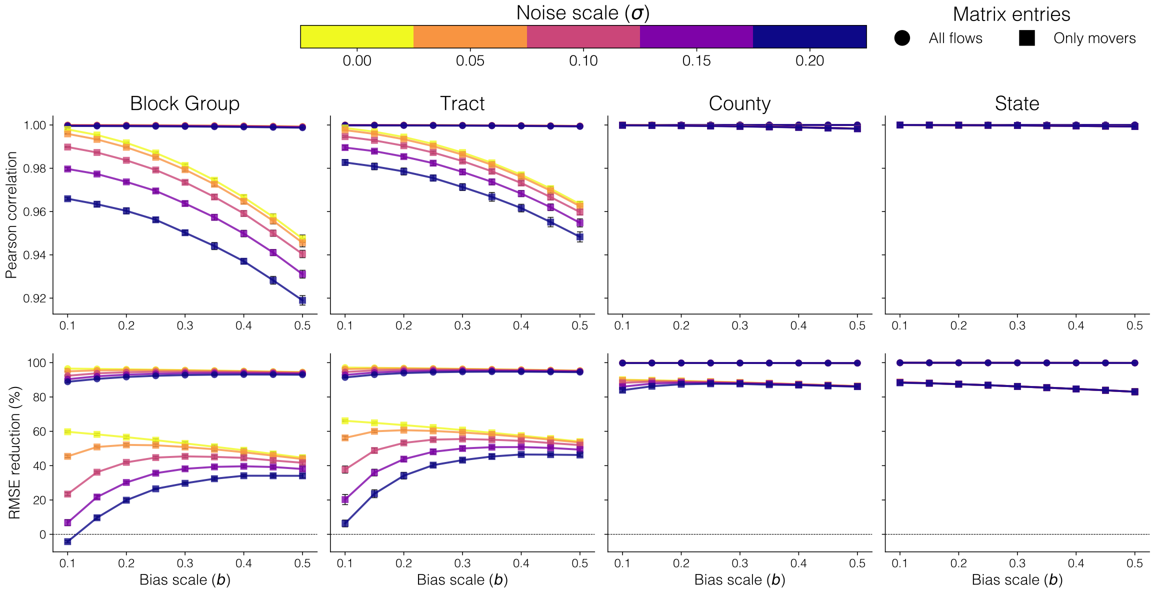

Supplementary Fig. 1: Pearson correlation and reduction in RMSE achieved by our harmonization procedure on semi-synthetic data with real-world bias and independent noise. We report average metrics across all years; error bars plot standard deviation across years (from 2010-11 to 2018-19, $n = 9$). Circles represent metrics computed on all entries of the flow matrix; squares represent metrics computed on movers only (off-diagonal elements). Columns assess metrics for flows at the CBG level, Census Tract level, county level, and state level. We assess results when varying the level of bias (x-axis) and independent noise (line color).

Supplementary Figure 1 plots the Pearson correlation and reduction in RMSE for flows at every geographic scale. We compute metrics with respect to all flows and movers only (off-diagonal entries) for varying levels of noise and bias. Results are consistent with the analysis above. When considering all flows (as opposed to just off-diagonal entries), error is reduced almost completely and correlations are nearly perfect at all geographic scales (circular points). When restricting to off-diagonal entries specifically (square points) on the more granular geographic scales (Census Tract and CBG levels), we find the largest error reductions when the scale of the independent noise is not too large relative to the bias, as expected. Pearson correlations decrease as bias and noise increase, but remain relatively high (above 0.91 in all cases). State and county-level metrics for movers remain robust at all noise and bias scales because we have better marginal data with which to correct them (including state-to-state movers and county populations).

Collectively, these synthetic experiments show that our harmonization procedure performs well in recovering the ground-truth matrix as long as the structure of the perturbation lies within the family it is able to estimate; in contrast, purely independent noise is unsurprisingly harder to correct

(both for our procedure and for any correction procedure). The family of perturbations our harmonization procedure can estimate is highly flexible (due to the number of scalings of varying shapes it incorporates) and indeed more flexible than classic iterative proportional fitting, which is widely used in similar demographic applications [4–8] and estimates only row and column-level scalings. The assumption that the perturbation is structured, as opposed to entirely independent for each entry, is also substantiated by our and previous findings of systematic demographic bias in Infutor data, which would not emerge under purely independent noise.

Related work documenting biases in the Infutor data

Many authors have assessed bias in the Infutor dataset, generally focusing on the data subsets or demographic characteristics most relevant to their own analyses. To our knowledge, the analysis we present in Figure 3 constitutes the first comprehensive documentation of biases in the Infutor dataset across (a) a long period of time and (b) the entire United States. We include here a non-exhaustive review of works with documentation of Infutor biases.

As we report in Table 2, Infutor undercounts the national US population. This is consistent with prior work; a similar finding is reported by Qian and Tan [9], Bernstein et al. [10], and Diamond et al. [11] among others. Phillips [12] additionally reports that, relative to 2010 ACS data, Infutor undercounts all state populations, but not by much, and out-of-state movers. A contrasting finding, reported by Diamond et al. [13], is that Infutor might overcount the population according to the 2000 Census in San Francisco. Such a finding might be driven by specific spatiotemporal idiosyncrasies of the San Francisco data in 2000, which is not included in the subset we analyze; however, the bulk of the literature, and all the literature which analyzes the same subset of the data we do, is consistent with our finding that Infutor undercounts the data. Diamond et al. [13] also suggest that Infutor might overcount populations due to lacking information about deaths; this is consistent with our finding that Infutor overcounts the older populations in Figure 3**c**.

In Figure 3, we also assess whether under- or over-represents demographic subpopulations. (Prior to conducting this assessment, we scale the Infutor population to match the overall population, so any discrepancies are not caused merely by overall undercounting.) Ramiller et al. [14] similarly study representation biases of Infutor in King County, Washington, during the 2015-2019 5-year period. They find that Infutor undercounts the county population while overrepresenting White, older, home-owning, and high-income populations. Later, they regress population errors in tract-level demographics to find that Infutor records are overrepresented in areas with higher White and high-income populations among other characteristics. We confirm most of their findings in this limited spatiotemporal scale at national level. However, the authors use the NARC3 dataset—a subset of Infutor's address records that contains detailed demographic information—and thus, beyond the spatiotemporal scope, our analyses might be substantially different.

Assessment of additional demographic biases

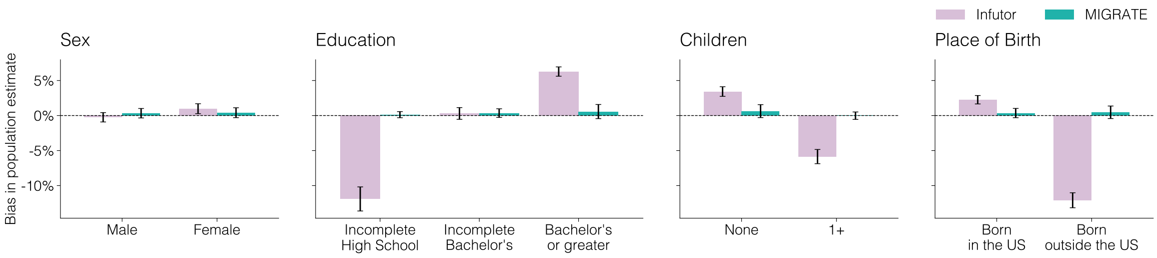

Supplementary Fig. 2: Additional analysis of socioeconomic and demographic biases at CBG population level. Variables are taken from the 5-year ACS CBG estimates. Bias is averaged over $n = 5$ population data releases (American Community Survey 5-year estimates, 2015 through 2018) Error bars represent standard deviations across these releases.

We documented biases in the Infutor dataset geographically (Figure 3**a**) and along the dimensions of urbanization, age, race, household tenure, and poverty (Figure 3**c**). Here, we provide additional analyses of demographic bias by sex, educational attainment, number of children per household,

and country of birth (Supplementary Figure 2). We find that Infutor displays negligible bias on the basis of sex. However, Infutor underrepresents populations with less formal education while overrepresenting populations with more formal education. Populations living in households with no children are overrepresented while those in households with children are underrepresented. This finding is consistent with the Infutor data documentation, which describes a lack of records for people under the age of 18 (therefore, children will be undercounted). Infutor also undercounts foreign-born residents by about 12%. MIGRATE essentially eliminates biases along all these demographic dimensions.

Biases in Representation of Immigrant Populations

Because of our finding that Infutor undercounts the foreign-born population, and previous research hypothesizing that Infutor may undercount undocumented immigrants specifically [10,12,15], we conduct further analyses investigating Infutor's representation of undocumented immigrants. Infutor draws from many data sources — including cell phone bills, phone books, credit header files, public government records, property deeds, county property records, vehicle warranties, and data from vehicle repair and maintenance providers [11,16,17] — and some of these sources may contain data for undocumented immigrants. There were an estimated 11 million undocumented immigrants in the United States in the 2018-2022 period, about 27% of the estimated foreign-born population [18].

To measure the biases of Infutor in representing the undocumented immigrant population, we investigate whether the relative population error correlates with estimates of the undocumented population. We use data on the yearly undocumented population per state provided by the Pew Research Center [19], which is the most granular data available. As expected from the results in Figure 3**a**, which documents bias in Infutor data at the county level, there is geographic bias in the Infutor data at the state level as well. Infutor undercounts the population in states with larger proportions of undocumented immigrants: there is a Spearman correlation of $\rho = -0.401$ between the state-level Infutor error and the proportion of undocumented immigrants per state, averaging across the decade (Supplementary Figure 3**b**).

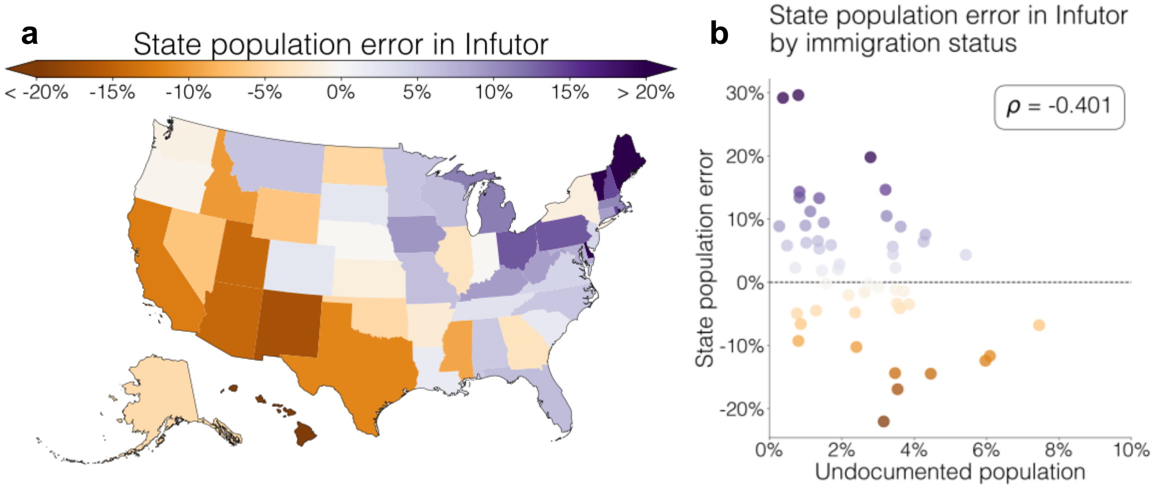

Supplementary Fig. 3: Infutor undercounts the undocumented immigrant population. We study the representation of the undocumented immigrant population by Infutor at the state level. **(a)** Average errors in state populations in Infutor data relative to Census data. MIGRATE estimates remove all state-level errors by construction. **(b)** Spearman correlation between the estimated share of undocumented immigrants in the state population (x-axis) and error in Infutor population estimates (y-axis). Infutor is more likely to undercount states with larger proportions of undocumented immigrants.

Importantly, as with other demographic biases in the Infutor dataset, harmonizing the dataset with Census data reduces biases with respect to immigrant populations as well. Census statistics do include undocumented immigrants [20], and so rescaling to match Census populations will mitigate

Infutor's biases.[1] In particular, MIGRATE corrects all bias at state level by construction. As shown in Supplementary Figure 2, MIGRATE also significantly reduces the bias for the overall foreign-born population.

[1]However, Census data may also be imperfect with respect to its coverage of undocumented migrants if they are, for example, less likely to respond to surveys.

Additional analyses of national migration

In this section we provide additional analyses of national migration trends.

Out-migration rates by CBG type

		Out-migration rate (per 1,000)	Out-movers
**Plurality Race**	White	132	31,425,400
	Black	128	3,823,677
	Asian	124	889,755
	Hispanic	118	5,289,710
**Urbanization**	Urban	135	35,481,142
	Rural	104	5,947,401
**Income Quartile**	Bottom	130	8,621,155
	2nd	130	9,944,217
	3rd	131	11,011,339
	Top	128	11,851,831

Supplementary Table 2: Out-migration rates (per 1,000 people) and raw counts of out-movers categorized by plurality race, urbanization level, and income quartile. We average the values across all years in the period.

Supplementary Table 2 reports the average out-migration rates for each of the ten CBG groups we analyze in the main text. The average out-migration rate in all CBGs is 13.0%, with 0.9% standard deviation across years. Out-migration rates are lower in plurality Hispanic and rural CBGs; out-migration rates remain similar across income quartiles.

Additional statistics on migration homophily

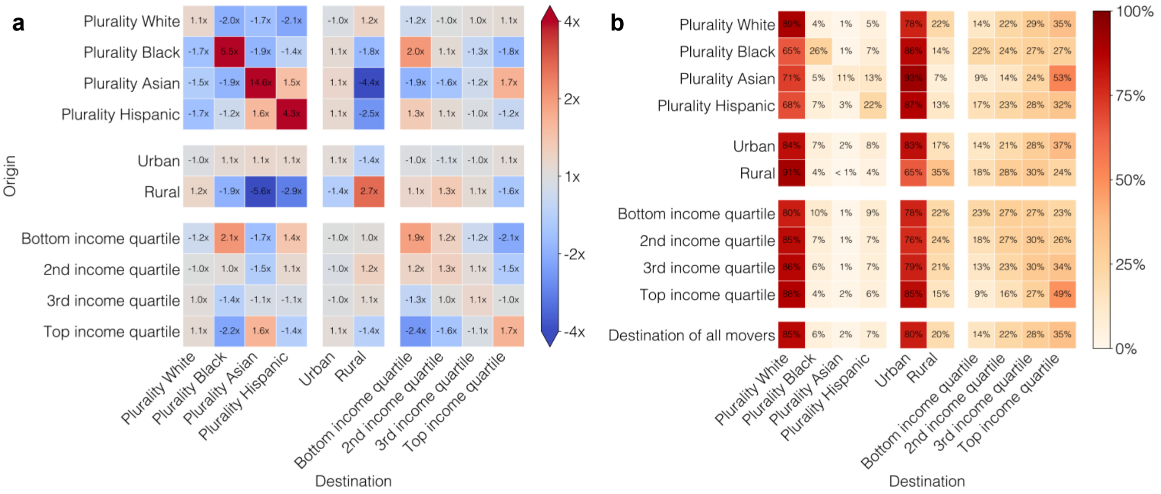

Supplementary Fig. 4: Additional national migration statistics. **(a)** Flows between the ten types of CBGs discussed in the main text relative to the share of all movers who moved to CBGs in the particular group. Each cell reports the ratio $\frac{\text{share of movers from origin CBG group moving to destination CBG group}}{\text{share of all movers moving to destination CBG group}}$; for example, the top left value of 1.1× reports the ratio of the share of movers from plurality white CBGs who move to plurality white CBGs (90%) to the share of movers from all CBGs who move to plurality white CBGs (80%). **(b)** Flows between the ten types of CBGs discussed in the main text when restricting to out-of-county movers demonstrate that homophily persists for long-distance moves.

In Supplementary Figure 4, we extend the analysis of national homophily patterns in migration documented in Figure 4**a**. Supplementary Figure 4**a** shows the shares of movers moving between groups of CBGs relative to the share of all movers who move to that group of CBGs (that is, a

version of Figure 4**a** where the value in each cell is divided by the corresponding value in the row 'Movers from all CBGs').

Homophily in migration might occur because many moves are local (within county or within 5 miles) and demographics are spatially correlated. To investigate this possibility, we restrict our analysis to out-of-county moves in Figure Supplementary 4**b**. Absolute racial homophily is less prominent—i.e. there are smaller shares in the diagonal of the race by race submatrix—yet still present: out-of-county movers from plurality Black, Asian, and Hispanic CBGs are 4.3×, 5.5×, and 3.1× likelier than out-of-county movers as a whole to move to CBGs with the same plurality race group (vs. 5.5×, 14.6×, and 4.3× for all movers).

Additional statistics on economic mobility in migration

Supplementary Figure 5 provides additional analyses supporting our finding of racial disparities in economic mobility (Figure 4b): movers from plurality Asian CBGs are more likely than movers as a whole, and movers from plurality Black CBGs less likely, to move to higher-income CBGs, even when controlling for income of origin CBG. We show that analogous racial disparities still emerge when looking at probability of moving to a CBG in the top income quartile (Supplementary Figure 5**a**) or bottom income quartile (Supplementary Figure 5**b**) as opposed to the probability of moving to a higher-income CBG (as in the main text). These disparities also emerge when controlling for origin

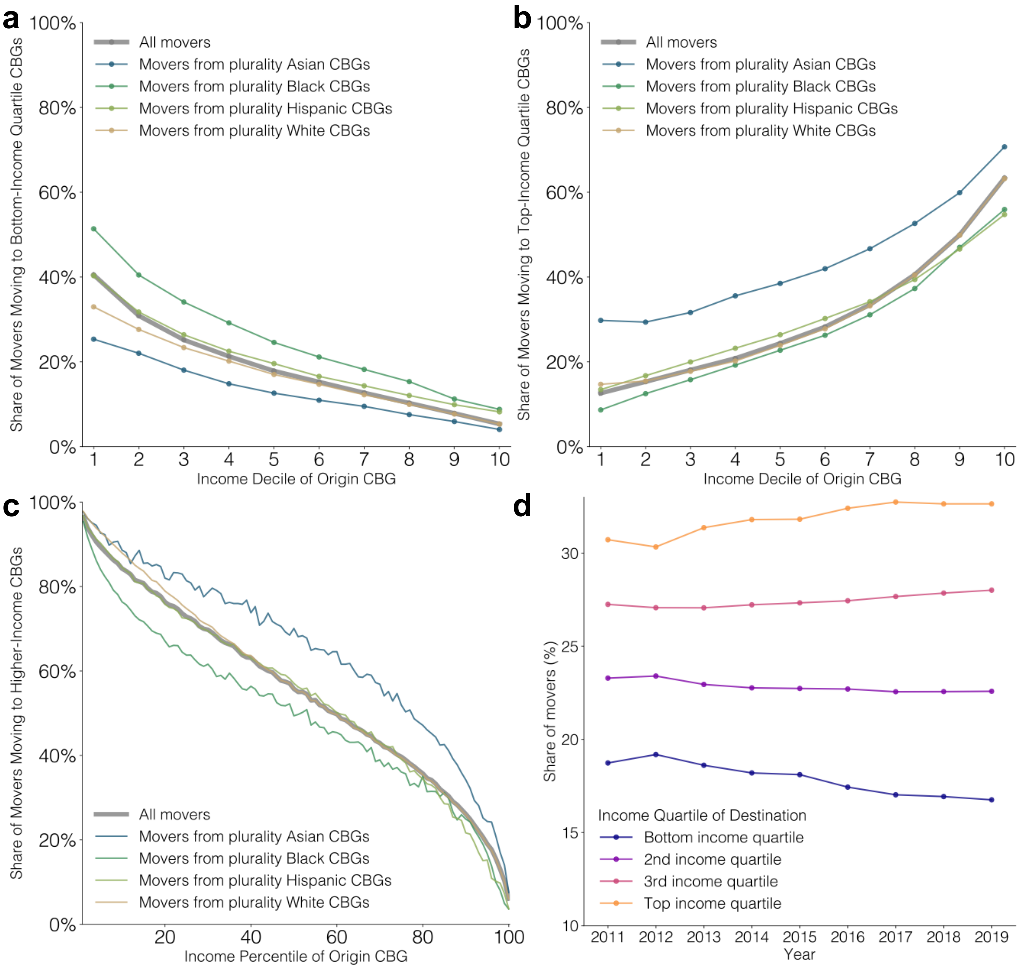

Supplementary Fig. 5: Further national migration statistics on upwards mobility. Probability of moving to a **(a)** top-income quartile CBG, **(b)** bottom-income quartile CBG, and **(c)** higher-income CBG conditioned on current residence median income and plurality race. In **(c)**, we classify current residence CBGs by income percentiles as opposed to deciles (as in the main text). Disparities remain robust. **(d)** Movers remain more likely to move to top-income-quartile CBGs than bottom-income-quartile CBGs throughout the decade, with the disparity increasing in magnitude over time.

CBG income percentile (Supplementary Figure 5**c**), as opposed to decile, as in the main text. We also observe that the gap in upward mobility widens during the decade: the share of movers going to a top income quartile CBG increases from 31% to 33% while the share of movers going to a bottom income quartile CBG decreases from 19% to 17% (Supplementary Figure 5**d**); overall, the ratio of out-movers moving to top income quartile versus bottom income quartile CBGs increases from 1.64 to 1.95.

Additional statistics on moving distance

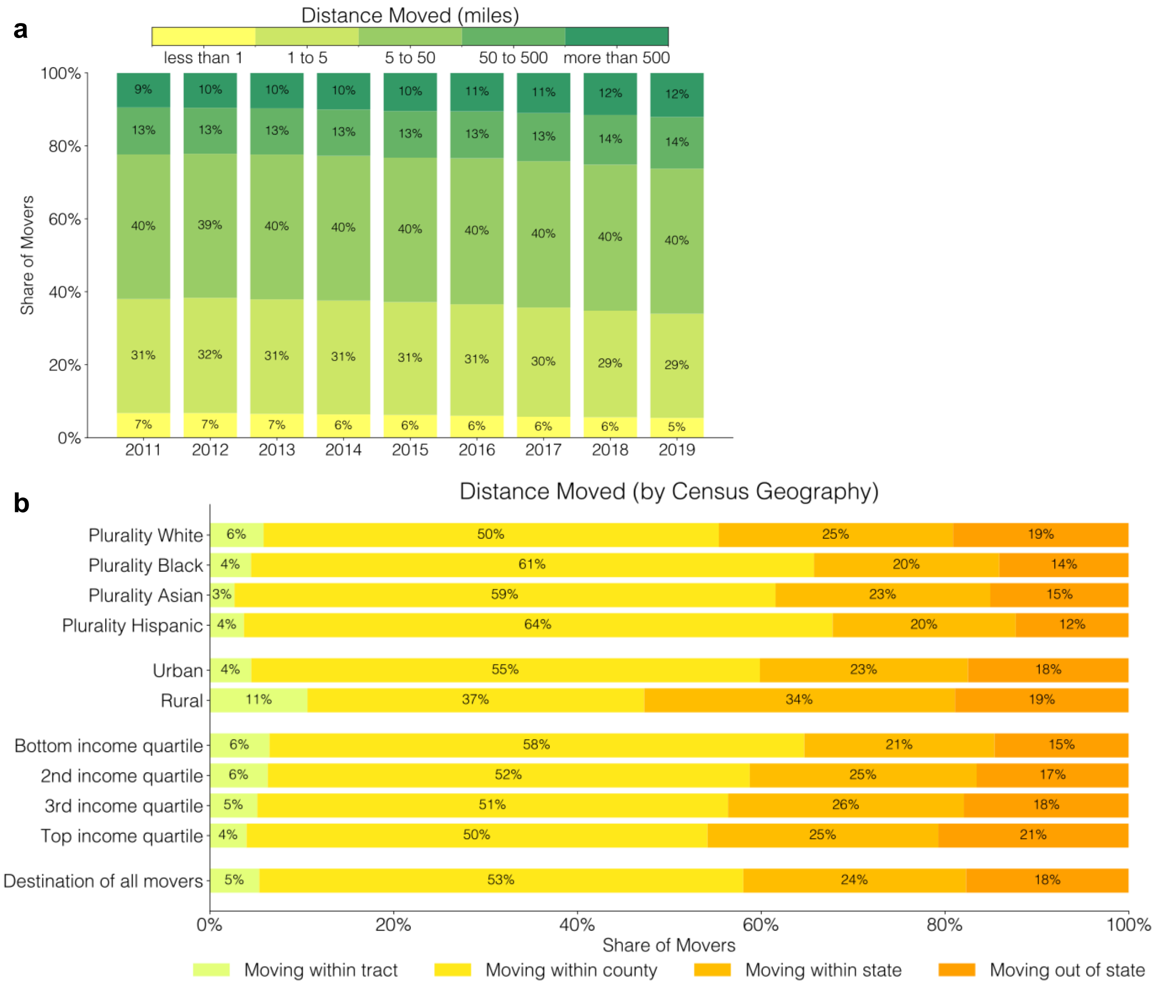

Supplementary Fig. 6: Further national migration statistics on distance moved. **(a)** Moving distance by year. **(b)** Fraction of movers moving within tract, county, and state.

Supplementary Figure 6 provides additional statistics on moving distance, expanding the findings in Figure 4**c**. Supplementary Figure 6**a** reports how moving distance changes over time, showing a general increase over the decade: the share of movers moving over 50 miles, for example, grows from about 22% to about 26%, and the average moving distance increases from 154 to 191 miles in the decade. Supplementary Figure 6**b** reports moving distance stratified by geographic boundary (that is, the share of movers remaining within tract, county, and state). Findings are generally consistent with those reported in Figure 4**c**, and also with published ACS figures, which state that over half of moves happen within counties[21].

Additional case studies of local migration patterns

In this section we provide two case studies of how MIGRATE reveals policy-relevant local migration patterns absent from publicly available data in New York City: a study of socioeconomic disparities on out-mover destinations, and a description of migration patterns to and from public housing buildings.

Case Study: destinations of New York City outmovers

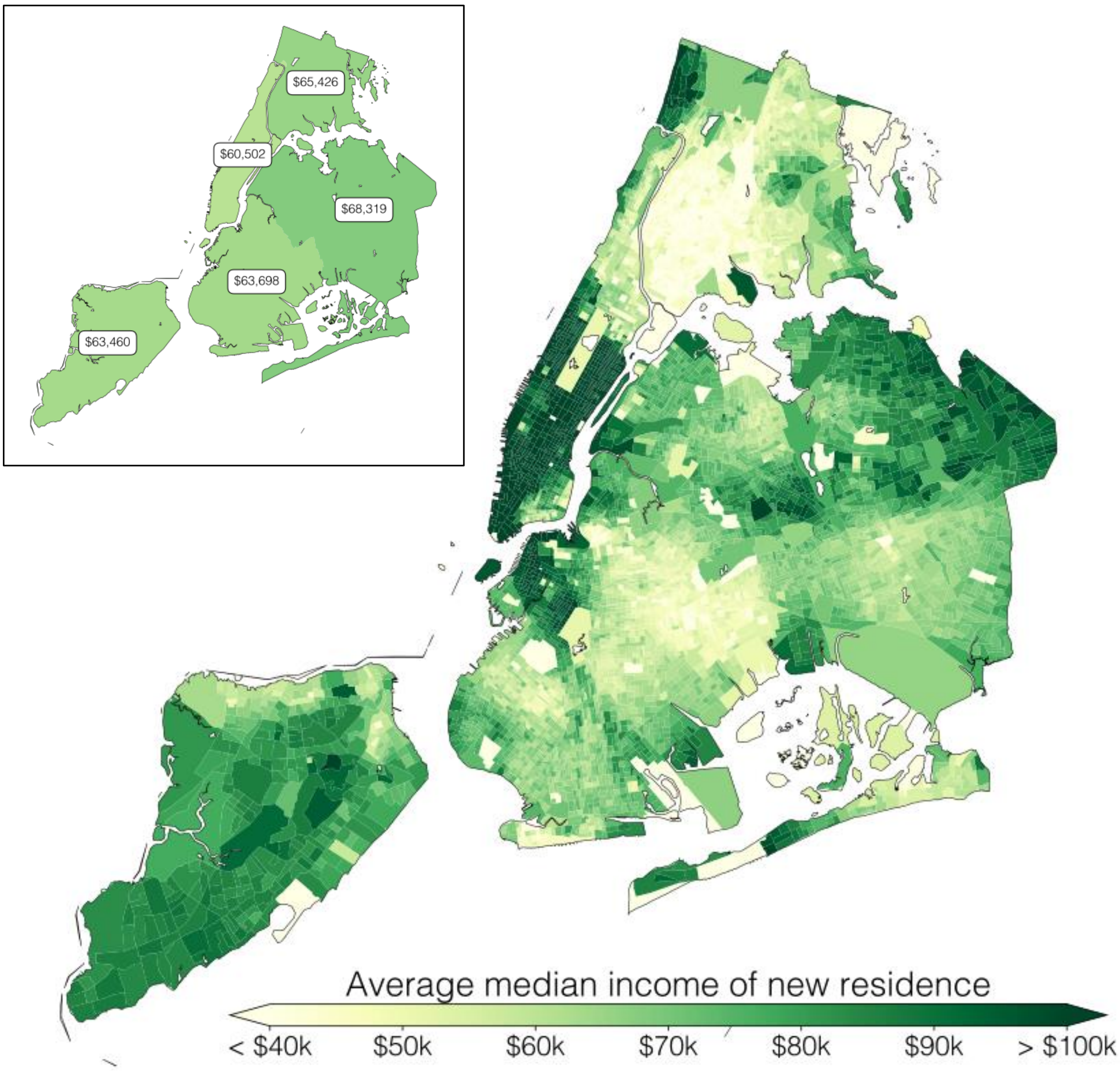

Supplementary Fig. 7: Average income (in US dollars $) of destination CBGs and counties for New York City out-movers in the 2010-19 period. The large map uses MIGRATE estimates and 5-year ACS CBG data. The inset map shows the corresponding distribution using only publicly available migration data from 5-year ACS county-to-county estimates.

We provide a case study contrasting MIGRATE with publicly available county-to-county migration data when analyzing migration stratified by a demographic or socioeconomic feature. Supplementary Figure 7 plots the average income of the CBG to which a person moves according to their previous CBG of residence. To compute this quantity for a given CBG i, we average the mean income of all destination CBGs j for out-movers of CBG i weighted by the number of out-movers from i to j. We perform an analogous computation for the county plot in the inset.

Comparing these results to the data available at county level reveals striking within-county variation. For example, the average income of the county to which people from Manhattan moved was $82,934—lower than that of the other four counties in New York City. MIGRATE reveals, however, that movers from some CBGs in Manhattan moved to areas with the highest average income among all New York City CBGs — above $175,000 at times — while movers from other Manhattan CBGs moved to areas with some of the lowest average incomes. Intra-county socioeconomic differences factor heavily in out-migration: Manhattan out-movers with the highest-income destinations tended to reside in the central and lower parts of the county (which tend to have higher incomes), whereas those with lower-income destinations came mostly from northern CBGs (which tend to have lower incomes). Similar within-county dynamics occur in the other four counties. Overall, this analysis illustrates the important demographic heterogeneity in migration which is concealed at the county level.

Case Study: New York City Housing Authority

Ensuring that residents have access to affordable housing is an important and challenging policy goal [22]. One way of achieving this is through the creation and management of public housing units: for example, the New York City Housing Authority (NYCHA) is the largest public housing authority in the US, providing affordable housing to over 500,000 authorized residents [23]. We provide a case study illustrating how the MIGRATE dataset can be used to understand which residents make use of public housing units, thereby illuminating the effect of this component of affordable housing policy.

We identify all 114 New York City Census Block Groups whose residential units are entirely owned by NYCHA using New York City's Primary Land Use Tax Lot Output (PLUTO) dataset [24] (Supplementary Figure 8**a**). We compare migration patterns for these CBGs, which we refer to as "NYCHA CBGs", to migration patterns of two other groups of non-NYCHA CBGs: (i) nearby CBGs (within 250 meters of a NYCHA development) and (ii) all non-NYCHA CBGs in New York City.[2] NYCHA CBGs have had consistently lower in-migration and out-migration rates than the two comparison groups of CBGs throughout the decade (Supplementary Figure 8**b**), implying a less mobile population. This is consistent with prior findings that residents of public housing and recipients of rental benefits have longer tenancies than average New York City residents [23,25].

To get a sense of the residents being served by NYCHA and their migration trajectories, we examine the demographics of the CBGs where NYCHA in-movers move from (Supplementary Figure 8**c**) and where NYCHA out-movers move to (Supplementary Figure 8**d**). Compared to non-NYCHA in-movers, NYCHA in-movers are likelier to move from plurality Black or Hispanic CBGs, as well as bottom income quartile CBGs. The same is true of NYCHA out-movers: they are also more likely to move to plurality Black or Hispanic CBGs, and bottom-income-quartile CBGs, than are non-NYCHA out-movers. The CBGs that NYCHA movers move to are higher-income than the CBGs they move from, providing evidence of upward mobility. NYCHA out-movers are also more upwardly mobile than non-NYCHA movers: 82% of NYCHA out-movers move to CBGs of higher median income, compared to 55% of all non-NYCHA out-movers.

We also quantify whether NYCHA in-movers and out-movers move within NYCHA, within New York City, and within New York State. There is a significant amount of shuffling within the NYCHA system: 14% of in-movers to a NYCHA CBG came from another NYCHA CBG, and 13% of out-movers from a NYCHA building go to another NYCHA CBG. This shuffling may be due in part to mandated renovations or building closures that result in temporary resident relocations by NYCHA [26–28]. On average across the decade that we study, NYCHA out-movers are likelier than non-NYCHA movers to remain within New York City (74% vs. 65%); however, this gap has decreased in recent years.

Overall, this analysis highlights how MIGRATE can provide insights into which residents are served by public housing policy. Beyond this case study of New York City, this approach could be extended to study public housing in other cities, or to study the effects of public housing foreclosures—which has been a topic of interest among other migration researchers [12].

[2]We excluded from the analysis all 345 CBGs (about 5.3% of all New York City CBGs) where some but not all of the residential units are owned by NCYHA.

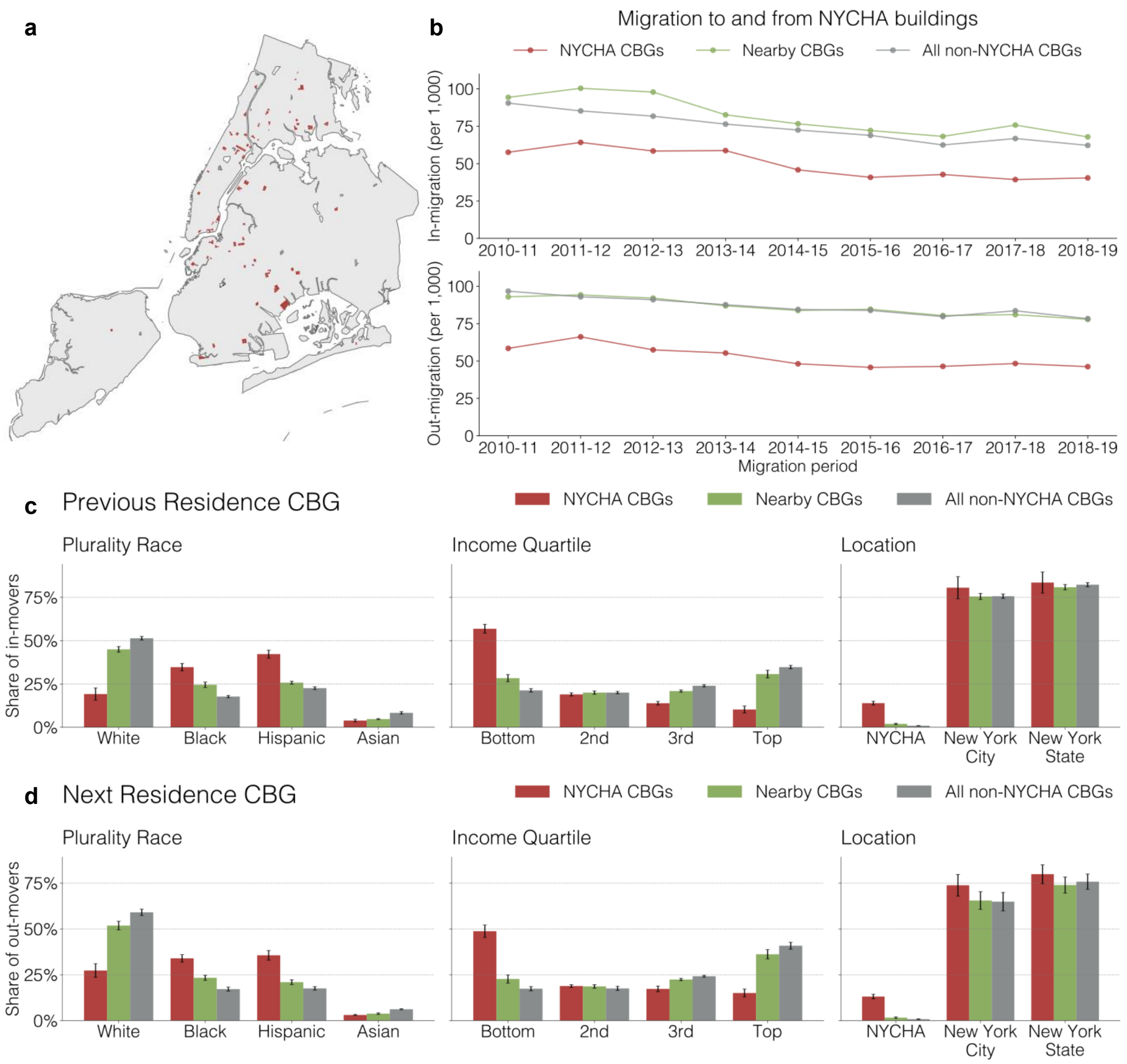

Supplementary Fig. 8: We use MIGRATE to understand migration to and from New York City Housing Authority (NYCHA) developments. **(a)** A map of the 114 Census Block Groups (marked in red) where all residential units are owned by NYCHA (i.e., where all the population lives in a NYCHA development). **(b)** Time series of in- and out-migration rates for NYCHA CBGs (red line), as well as for CBGs nearby (within 250 meters of a NYCHA development; green line) and non-NYCHA CBGs within New York City (gray line). NYCHA CBGs have consistently lower in- and out-migration rates, implying a less mobile population. **(c)** Demographics of previous residence CBG for in-movers into NYCHA buildings as well as for the other CBG groups considered, aggregated across the decade (error bars represent standard deviation across years). **(d)** Demographics of next residence CBG for out-movers.

Privacy Protections

In this section we detail the measures we took to preserve the privacy of individuals in the MIGRATE data release. Analysis of the Infutor dataset was determined to be not human subjects research by the Cornell Institutional Review Board (IRB #0145225).

First, in contrast to the original Infutor data, the data we release is aggregated: it contains only estimated migration between CBG-CBG pairs, and no information about individuals. This level of data aggregation is consistent with other data sources which have been made available to researchers. For example, mobility data often contains the aggregate counts of individuals moving from a given CBG to a given point of interest on a given day [7,29–32].

Second, the matrices we release are probabilistic and rescaled: in particular, the fact that a particular entry of the CBG-CBG migration matrix is non-zero does not guarantee that a given number of individuals moved between those CBGs (or indeed, that any did at all). This is because individuals are mapped probabilistically to CBGs (e.g., if we have only a ZIP code for an individual, they are mapped probabilistically to CBGs within that ZIP code) and the migration matrices are rescaled multiple times.

Third, we release the data only to researchers for non-profit use who agree to a data use agreement pledging not to re-identify individuals in the data, and to adhere to privacy-protecting measures when storing data and presenting results, following manual review of their application and research project.

Finally, we compute the average number of destinations (unique CBGs) for out-movers from each CBG. The purpose of the analysis is to assess how difficult it would be to track a given individual if one knew they lived in a given CBG and then moved. Across CBGs and years, the mean number of destinations for outmovers is 272.2 (median 234). We redact from the public data release migration information for the very small proportion of CBGs (0.558%) for which 90% of out-movers travel to 10 or fewer CBGs. All analyses in the paper were conducted with the full data.

References

[1] Balachandar, S., Garg, N., Pierson, E.: Domain constraints improve risk prediction when outcome data is missing. In: The Twelfth International Conference on Learning Representations (2024). https://openreview.net/forum?id=1mNFsbvo2P

[2] Agostini, G., Pierson, E., Garg, N.: A bayesian spatial model to correct under-reporting in urban crowdsourcing. In: Proceedings of the AAAI Conference on Artificial Intelligence, vol. 38, pp. 21888–21896 (2024)

[3] Pierson, E., Koh, P.W., Hashimoto, T., Koller, D., Leskovec, J., Eriksson, N., Liang, P.: Inferring multidimensional rates of aging from cross-sectional data. In: The 22nd International Conference on Artificial Intelligence and Statistics, pp. 97–107 (2019). PMLR

[4] Birkin, M., Clarke, M.: Synthesis—A Synthetic Spatial Information System for Urban and Regional Analysis: Methods and Examples. Environment and Planning A: Economy and Space **20**(12), 1645–1671 (1988) https://doi.org/10.1068/a201645 . Publisher: SAGE Publications Ltd. Accessed 2025-09-03

[5] Wong, D.W.S.: The Reliability of Using the Iterative Proportional Fitting Procedure. The Professional Geographer **44**(3), 340–348 (1992) https://doi.org/10.1111/j.0033-0124.1992.00340.x . _eprint: https://onlinelibrary.wiley.com/doi/pdf/10.1111/j.0033-0124.1992.00340.x. Accessed 2025-09-03

[6] Simpson, L., Tranmer, M.: Combining Sample and Census Data in Small Area Estimates: Iterative Proportional Fitting with Standard Software. The Professional Geographer **57**(2), 222–234 (2005) https://doi.org/10.1111/j.0033-0124.2005.00474.x . _eprint: https://onlinelibrary.wiley.com/doi/pdf/10.1111/j.0033-0124.2005.00474.x. Accessed 2025-09-03

[7] Chang, S., Pierson, E., Koh, P.W., Gerardin, J., Redbird, B., Grusky, D., Leskovec, J.: Mobility network models of covid-19 explain inequities and inform reopening. Nature **589**(7840), 82–87 (2021)

[8] Chang, S., Koehler, F., Qu, Z., Leskovec, J., Ugander, J.: Inferring dynamic networks from marginals with iterative proportional fitting. ICML (2024)

[9] Qian, F., Tan, R.: The effects of high-skilled firm entry on incumbent residents. Stanford Institute for Economic Policy Research (SIEPR) Working Paper, 21–039 (2021)

[10] Bernstein, S., Diamond, R., Jiranaphawiboon, A., McQuade, T., Pousada, B.: The contribution of high-skilled immigrants to innovation in the united states. Technical report, National Bureau of Economic Research (2022)

[11] Diamond, R., McQuade, T., Qian, F.: The Effects of Rent Control Expansion on Tenants, Landlords, and Inequality: Evidence from San Francisco. American Economic Review **109**(9), 3365–3394 (2019) https://doi.org/10.1257/aer.20181289 . Accessed 2025-01-24

[12] Phillips, D.C.: Measuring Housing Stability With Consumer Reference Data. Demography **57**(4), 1323–1344 (2020) https://doi.org/10.1007/s13524-020-00893-5

[13] Diamond, R., Guren, A., Tan, R.: The Effect of Foreclosures on Homeowners, Tenants, and Landlords. National Bureau of Economic Research (2020). https://doi.org/10.3386/w27358 . https://www.nber.org/papers/w27358 Accessed 2025-01-24

[14] Ramiller, A., Song, T., Parker, M., Chapple, K.: Residential Mobility and Big Data: Assessing the Validity of Consumer Reference Datasets. Cityscape **26**(3), 227–240 (2024). Publisher: US Department of Housing and Urban Development. Accessed 2025-01-24

[15] Downes, H., Zuo, G.: Can moves to opportunity be constructed? evidence from the low-income

housing tax credit. In: 2023 APPAM Fall Research Conference (2023). APPAM

[16] Phillips, D.C., Sullivan, J.X.: Personalizing homelessness prevention: Evidence from a randomized controlled trial. Journal of Policy Analysis and Management **43**(4), 1101–1128 (2024) https://doi.org/10.1002/pam.22547 https://onlinelibrary.wiley.com/doi/pdf/10.1002/pam.22547

[17] Boar, C., Giannone, E.: Consumption segregation. Technical report, National Bureau of Economic Research (2023)

[18] Baker, B., Warren, R.: Estimates of the Unauthorized Immigrant Population Residing in the United States: January 2018–January 2022. Office of Homeland Security Statistics (2024). https://ohss.dhs.gov/topics/immigration/unauthorized/population-estimates Accessed 2025-08-07

[19] Passel, J., Krogstad, J.: What we know about unauthorized immigrants living in the US (2024). https://www.pewresearch.org/short-reads/2024/07/22/what-we-know-about-unauthorized-immigrants-living-in-the-us Accessed 2025-08-15

[20] US Census Bureau: About the Foreign-Born Population. Section: Government (2025). https://www.census.gov/topics/population/foreign-born/about.html Accessed 2025-08-07

[21] Kerns-D'Amore, K., McKenzie, B., Locklear, L.S.: Migration in the United States: 2006 to 2019. Technical Report ACS-53, American Community Survey (July 2023). https://www.census.gov/content/dam/Census/library/publications/2023/acs/acs-53.pdf

[22] Favilukis, J., Mabille, P., Van Nieuwerburgh, S.: Affordable housing and city welfare. The Review of Economic Studies **90**(1), 293–330 (2023)

[23] New York City Housing Authority: NYCHA Fact Sheet. Accessed: 2025-05-06 (2024). https://www.nyc.gov/assets/nycha/downloads/pdf/NYCHA_Fact_Sheet.pdf

[24] New York City Department of City Planning: Primary Land Use Tax Lot Output (PLUTO). https://www.nyc.gov/site/planning/data-maps/open-data/dwn-pluto-mappluto.page. Accessed: 2025-05-04 (2024)

[25] Chen, R., Jiang, H., Quintero, L.E.: Measuring the value of rent stabilization and understanding its implications for racial inequality: Evidence from New York City. Regional Science and Urban Economics **103**, 103948 (2023) https://doi.org/10.1016/j.regsciurbeco.2023.103948 . Accessed 2025-05-06

[26] Navarro, M.: Harlem Housing Relic From the 1800s Is Set for a Long-Promised Overhaul. The New York Times (2014). Chap. New York. Accessed 2025-05-06

[27] Fernandez, M.: New York Plans to Topple Public Housing Towers. The New York Times (2010). Chap. New York. Accessed 2025-05-06

[28] Zaveri, M.: To Improve Public Housing, New York City Moves to Tear It Down. The New York Times (2023). Chap. New York. Accessed 2025-05-06

[29] Abbiasov, T., Heine, C., Sabouri, S., Salazar-Miranda, A., Santi, P., Glaeser, E., Ratti, C.: The 15-minute city quantified using human mobility data. Nature Human Behaviour **8**(3), 445–455 (2024)

[30] Kostandova, N., Schluth, C., Arambepola, R., Atuhaire, F., Bérubé, S., Chin, T., Cleary, E., Cortes-Azuero, O., García-Carreras, B., Grantz, K.H., *et al.*: A systematic review of using population-level human mobility data to understand sars-cov-2 transmission. Nature Communications **15**(1), 1–12 (2024)

[31] Xu, F., Wang, Q., Moro, E., Chen, L., Salazar Miranda, A., González, M.C., Tizzoni, M., Song, C., Ratti, C., Bettencourt, L., et al.: Using human mobility data to quantify experienced urban inequalities. Nature Human Behaviour, 1–11 (2025)

[32] Yabe, T., García Bulle Bueno, B., Frank, M.R., Pentland, A., Moro, E.: Behaviour-based dependency networks between places shape urban economic resilience. Nature Human Behaviour, 1–11 (2024)
