# Supplementary material for: Inferring fine-grained migration patterns across the United States
Source: Nat Commun. 2025 Dec 26;17:1265. doi: 10.1038/s41467-025-68019-2 (PMC12868740; doi:10.1038/s41467-025-68019-2)
Supplement: Supplementary file 2 — Reporting Summary [file 41467_2025_68019_MOESM2_ESM.pdf]

nature portfolio

Corresponding author(s): Emma Pierson, Gabriel Agostini

Last updated by author(s): Nov 16, 2025

Reporting Summary

Nature Portfolio wishes to improve the reproducibility of the work that we publish. This form provides structure for consistency and transparency in reporting. For further information on Nature Portfolio policies, see our Editorial Policies and the Editorial Policy Checklist.

Statistics

For all statistical analyses, confirm that the following items are present in the figure legend, table legend, main text, or Methods section.

n/a	Confirmed	
☐	☒	The exact sample size (n) for each experimental group/condition, given as a discrete number and unit of measurement
☐	☒	A statement on whether measurements were taken from distinct samples or whether the same sample was measured repeatedly
☐	☒	The statistical test(s) used AND whether they are one- or two-sided<br>*Only common tests should be described solely by name; describe more complex techniques in the Methods section.*
☐	☒	A description of all covariates tested
☒	☐	A description of any assumptions or corrections, such as tests of normality and adjustment for multiple comparisons
☐	☒	A full description of the statistical parameters including central tendency (e.g. means) or other basic estimates (e.g. regression coefficient) AND variation (e.g. standard deviation) or associated estimates of uncertainty (e.g. confidence intervals)
☒	☐	For null hypothesis testing, the test statistic (e.g. F, t, r) with confidence intervals, effect sizes, degrees of freedom and P value noted<br>*Give P values as exact values whenever suitable.*
☒	☐	For Bayesian analysis, information on the choice of priors and Markov chain Monte Carlo settings
☒	☐	For hierarchical and complex designs, identification of the appropriate level for tests and full reporting of outcomes
☐	☒	Estimates of effect sizes (e.g. Cohen's d, Pearson's r), indicating how they were calculated

Our web collection on statistics for biologists contains articles on many of the points above.

Software and code

Policy information about availability of computer code

Data collection	All code used to collect data is available via GitHub: https://github.com/gsagostini/MIGRATE/tree/main
Data analysis	All code used to analyze data is available via GitHub: https://github.com/gsagostini/MIGRATE/tree/main

For manuscripts utilizing custom algorithms or software that are central to the research but not yet described in published literature, software must be made available to editors and reviewers. We strongly encourage code deposition in a community repository (e.g. GitHub). See the Nature Portfolio guidelines for submitting code & software for further information.

Data

Policy information about availability of data

All manuscripts must include a data availability statement. This statement should provide the following information, where applicable:

- Accession codes, unique identifiers, or web links for publicly available datasets
- A description of any restrictions on data availability
- For clinical datasets or third party data, please ensure that the statement adheres to our policy

The data we produce (MIGRATE) is available upon request for non-profit research use at migrate.cornell.tech.edu (hosted on Zenodo, with doi doi.org/10.48550/arXiv.2503.20989). To mitigate any privacy risks, interested researchers must agree to a data usage agreement pledging not to re-identify individuals in the data, and to adhere to privacy-protecting measures when storing data and presenting results. Manual review of their application should be completed within 10 business

days, and will last for the duration of the proposed research project. Census and ACS data, New York City tax lot data, and California fire perimeters are all publicly available. Raw address history data can be obtained from Infutor.

Research involving human participants, their data, or biological material

Policy information about studies with human participants or human data. See also policy information about sex, gender (identity/presentation), and sexual orientation and race, ethnicity and racism.

Reporting on sex and gender	We stratify migration patterns by Census area (block groups, tracts, counties, and states)). We do not analyze migration of any individual person, or analyze the sex or gender of any individual person. In a supplementary analysis we confirm that our migration patterns are unbiased with respect to the proportion of the Census area that is female, as derived from American Community Survey data.
Reporting on race, ethnicity, or other socially relevant groupings	As mentioned above, we stratify migration patterns by Census area (block groups, tracts, counties, and states). We do not analyze migration of any individual person, or analyze the race or other demographic attribute of any individual person. We confirm that our migration patterns are unbiased with respect to demographic attributes (Figure 3 and Figure S2) including age, sex, education, urban/rural status, poverty level, race, owner/renter status, and presence of children in the household. We also analyze how migration patterns differ across 10 types of Census block groups: plurality white, Asian, Black, and Hispanic; urban versus rural; and bottom, second, third, and top income quartile. In all cases, demographic data are derived from American Community Survey data for the relevant Census areas.
Population characteristics	This research does not rely on human research participants.
Recruitment	See above.
Ethics oversight	Analysis of the Infutor dataset was determined to be not human subjects research by the Cornell Institutional Review Board (IRB #0145225).

Note that full information on the approval of the study protocol must also be provided in the manuscript.

Field-specific reporting

Please select the one below that is the best fit for your research. If you are not sure, read the appropriate sections before making your selection.

☐ Life sciences ☒ Behavioural & social sciences ☐ Ecological, evolutionary & environmental sciences

For a reference copy of the document with all sections, see nature.com/documents/nr-reporting-summary-flat.pdf

Behavioural & social sciences study design

All studies must disclose on these points even when the disclosure is negative.

Study description	Quantitative analysis of US migration data.
Research sample	The research sample comes from the following existing datasets:<br>1. Address histories from the company Infutor (https://batchdocs.infutor.com/#doc-section-1) collected from data sources including voter files, property deeds, credit files, and phone books. We do not use any demographic information from the Infutor dataset, and representativity of the dataset is the object of study of our paper.<br>2. Data from the US Census (https://data.census.gov/)<br>3. Data from the American Community Survey (https://www.census.gov/programs-surveys/acs.html)
Sampling strategy	Infutor collects data on the US population from data sources including voter files, property deeds, credit files, and phone books. The US Census counts every individual currently residing in the United States, and the American Community Survey takes an unbiased sample of the same population.
Data collection	The researchers did not collect the data themselves.
Timing	The data analyzed was collected between 2010 and 2019.
Data exclusions	Data from US territories was excluded from the analysis due to lack of consistent ACS and Census data; these exclusions amount to 0.26% of all unique address records in the raw Infutor dataset. Additionally, 0.53% of the unique address records were excluded because they could not be mapped to an existing Census Block Group in the United States. The Methods section provides further details.
Non-participation	No participants dropped out/declined participation
Randomization	Participants were not allocated into experimental groups.

Reporting for specific materials, systems and methods

We require information from authors about some types of materials, experimental systems and methods used in many studies. Here, indicate whether each material, system or method listed is relevant to your study. If you are not sure if a list item applies to your research, read the appropriate section before selecting a response.

Materials & experimental systems

n/a	Involved in the study
☒	☐ Antibodies
☒	☐ Eukaryotic cell lines
☒	☐ Palaeontology and archaeology
☒	☐ Animals and other organisms
☒	☐ Clinical data
☒	☐ Dual use research of concern
☒	☐ Plants

Methods

n/a	Involved in the study
☒	☐ ChIP-seq
☒	☐ Flow cytometry
☒	☐ MRI-based neuroimaging

Plants

Seed stocks	The research does not involve plants.
Novel plant genotypes	The research does not involve plants.
Authentication	The research does not involve plants.
